# Differences in effectiveness and use of laparoscopic surgery in locally advanced colon cancer patients

**DOI:** 10.1038/s41598-021-89554-0

**Published:** 2021-05-11

**Authors:** M. Schootman, Matthew Mutch, T. Loux, J. M. Eberth, N. O. Davidson

**Affiliations:** 1 Department of Epidemiology and Biostatistics, College for Public Health and Social Justice, Saint Louis University, Saint Louis, MO USA; 2 Department of Clinical Analytics, SSM Health, 10101 Woodfield Lane, Saint Louis, MO USA; 3 Department of Surgery, Section of Colon and Rectal Surgery, Washington University School of Medicine, Saint Louis, MO USA; 4 Department of Epidemiology and Biostatistics, Arnold School of Public Health, University of South Carolina, Columbia, SC USA; 5 Division of Gastroenterology, Department of Medicine, Washington University School of Medicine, Saint Louis, MO USA; 6 Alvin J. Siteman Cancer Center At Barnes-Jewish Hospital, Washington University School of Medicine, Saint Louis, MO USA

**Keywords:** Colon cancer, Health care, Prognosis

## Abstract

Patients with locally advanced colon cancer have worse outcomes. Guidelines of various organizations are conflicting about the use of laparoscopic colectomy (LC) in locally advanced colon cancer. We determined whether patient outcomes of LC and open colectomy (OC) for locally advanced (T4) colon cancer are comparable in all colon cancer patients, T4a versus T4b patients, obese versus non-obese patients, and tumors located in the ascending, descending, and transverse colon. We used data from the 2013–2015 American College of Surgeons’ National Surgical Quality Improvement Program. Patients were diagnosed with nonmetastatic pT4 colon cancer, with or without obstruction, and underwent LC (n = 563) or OC (n = 807). We used a composite outcome score (mortality, readmission, re-operation, wound infection, bleeding transfusion, and prolonged postoperative ileus); length of stay; and length of operation. Patients undergoing LC exhibited a composite outcome score that was 9.5% lower (95% CI − 15.4; − 3.5) versus those undergoing OC. LC patients experienced a 11.3% reduction in postoperative ileus (95% CI − 16.0; − 6.5) and an average of 2 days shorter length of stay (95% CI − 2.9; − 1.0). Patients undergoing LC were in the operating room an average of 13.5 min longer (95% CI 1.5; 25.6). We found no evidence for treatment heterogeneity across subgroups (*p* > 0.05). Patients with locally advanced colon cancer who receive LC had better overall outcomes and shorter lengths of stay compared with OC patients. LC was equally effective in obese/nonobese patients, in T4a/T4b patients, and regardless of the location of the tumor.

## Introduction

Colorectal cancer (CRC) is a leading cause of cancer death with an estimated 95,520 new cases and 50,260 deaths in 2017^1^. Laparoscopic colectomy (LC) has gained widespread acceptance for resection of CRC, with better short term complication rates and similar long-term outcomes relative to open colectomy (OC) in stage I-III patients^2–4^. However, about ten percent of patients are diagnosed with locally advanced cancer (T4) with locoregional invasion, who may have worse outcomes^5^. Such tumors have a reasonable chance of a cure when accompanied by an en bloc multivisceral resection^6–8^, but a landmark randomized study excluded locally advanced colon cancer when comparing LC versus OC^9^.

The safety and effectiveness of LC in T4 colon cancer patients is still unresolved because of technical difficulties with en-bloc dissection of large tumors. The American Society of Colon & Rectal Surgeons recommends that “laparoscopic resection of T4 colon cancer may be performed safely and effectively with long-term oncologic outcomes that do not differ in comparison with open surgery.”^10^ In contrast, the National Comprehensive Cancer Network guidelines for colon cancer surgery recommend against LC when locally advanced disease is present^11^. The European Association of Endoscopic Surgery (EAES) also failed to recommend LC in locally advanced colon cancer^12^. While recent studies have compared LC with OC in locally advanced colon cancer^13–17^, many of those studies used small sample sizes or studied only patients from single institutions, while others were performed outside the United States.

There is also a major need to determine which T4 patients are appropriate candidates for LC^18^. For example, T4b tumors (invade and/or adhere to other organs or structures) are technically more demanding than T4a tumors (penetrate to the surface of the visceral peritoneum), depending on the nature of the multivisceral resection required to achieve negative margins. Resection of locally advanced tumors is difficult to perform laparoscopically because of the need to perform an extended resection and obtaining negative margins is more challenging^19^. The effectiveness of LC versus OC across different locations in locally advanced tumors in the colon is unknown. The advantages of LC have prompted its application to a wider range of patients with colon cancer such as those who are obese, have locally advanced tumors or require more extended resections. Older studies have argued that LC in obese patients is technically more difficult and could be a contraindication^20,21^. Additionally, observational studies with small patient samples examining transverse colon cancers have found that laparoscopic resection resulted in acceptable outcomes, but such studies may have been underpowered^22–24^. Studies to compare directly the effectiveness of LC versus OC across different locations in locally advanced tumors in the colon are not available, and transverse colon cancers were excluded from the landmark Clinical Outcomes of Surgical Therapy (COST) trial^9^.

We examined differences in 30 day outcomes of LC versus OC and also compared subgroups of patients: (1) T4a and T4b patients, (2) those with tumors located in the left, right, or transverse colon, and (3) obese and nonobese patients. Our findings should help guide the use of LC in minimally invasive surgery in T4 colon cancer patients.

## Materials and methods

### Data source

Data for our cohort was constructed using the 2013–2015 American College of Surgeons’ National Surgical Quality Improvement Program (ACS-NSQIP) Participant Use File and Targeted Colectomy File. Preoperative patient comorbidities, preoperative laboratory results, intraoperative procedures, 30-day postoperative mortality rates, and complications were abstracted by trained reviewers. Additional details are described here (https://www.facs.org/quality-programs/acs-nsqip). All methods were carried out in accordance with relevant guidelines and regulations. The Saint Louis University Institutional Review Board deemed this protocol to be exempt from oversight since it was nonhuman subjects research.

### Patient selection

All patients who underwent colectomy (with or without obstruction) and were diagnosed with pT4 colon cancer (and nonmetastatic disease M0 or Mx) were included. Patients who underwent LC, including robotic, or OC were included; patients with other types of surgery were excluded. We included patient in the LC groups that resulted in conversion to OC based on intention to treat^13,25^. Patients who underwent emergency surgery were excluded since they are much more likely to receive open surgery^26^, which may lead to difficulty creating comparable groups of OC and LC patients. To test the robustness of our findings, we performed a sensitivity analysis by including LC performed with open or hand assistance in the LC group. Hand or open assistance may be used as a bridge between a straight LC and an OC in cases where straight LC may be difficult because of the size of tumor, local advancement, or intra-abdominal adhesions^16^. There were no participants who were younger than 18 years of age.

### Patient outcomes

Outcomes included operation time, anastomotic leakage, mortality, readmission, re-operation, length of stay, wound infection, transfusion, prolonged postoperative ileus, sepsis, myocardial infarction, deep vein thrombosis, and pulmonary embolism. Conversion occurred when LC was converted to an unplanned OC. Re-operation was defined as any unplanned return to the operating room for a surgical procedure, for any reason, within 30 days of the colectomy at any hospital or surgical facility. All-cause mortality was defined as deaths within 30 days following surgery. Readmission was defined as any readmission (to the same or another hospital), for any reason, within 30 days of the colectomy. A wound infection included superficial or deep incisional surgical site infection or any other wound infection. Postoperative bleeding occurrence was defined as any transfusion given from the start of the colectomy to 72 h post operation. Because of the infrequent occurrence for some of the postoperative outcomes, we constructed a composite outcome of mortality, readmission, re-operation, wound infection, bleeding occurrence, and prolonged postoperative ileus. Patients with at least one adverse outcome were contrasted to those without any such adverse outcomes^27^, recognizing the variability in the severity of its components but summarizing information across several quality dimensions.

### Covariates

Based on previous studies^28,29^, we included patient sex, age, Hispanic ethnicity, race, ASA grouping, smoking status, chronic comorbid conditions (congestive heart failure, hypertension, chronic obstructive pulmonary disease, chronic kidney disease, anemia, weight loss > 10%, bleeding disorder, transfusion), patient functional status prior to surgery, chemotherapy, and mechanical bowel preparation.

T4a and T4b colon cancers were determined based on AJCC TNM staging. Tumor location was based on post-operative diagnosis using ICD-9 or ICD-10 codes. The patient’s most recent height and weight documented in the medical record within the 30 days prior to the colectomy or at the time the patient was being considered a candidate for surgery was used to calculate BMI. Patients were classified as obese (BMI ≥ 30 kg/m^2^) or nonobese (BMI < 30 kg/m^2^).

### Statistical analysis

We used 1:1 propensity score matching to obtain patient groups with comparable characteristics to assess the effect of LC on patient outcomes with all covariates. We calculated standardized differences for all covariates before and after matching. A standardized difference of less than − 0.1 or greater than 0.1 was used as a marker for imbalance^30^. We also compared the means and variances of the covariates after matching to determine the balance between the LC and OC groups. We calculated the average treatment effect among the treated (ATT) and associated 95% confidence intervals to describe the effectiveness of LC versus OC, focusing on absolute differences.

To assess potential heterogeneous treatment effects of LC by T4 type, we first matched patients who received LC to those who received OC. Matching was done in a 2:1 ratio using optimal matching on the propensity score, e(x), with a caliper of 0.25 × sd(e[x])^31^. Once patients were matched, we ran separate regressions in the LC and OC groups to predict respective outcomes. The coefficients for T4 type were compared between the two models to assess treatment heterogeneity due to these factors^32^. A similar approach was used to assess treatment heterogeneity due to tumor location (left, right, transverse) and obesity.

We conducted sensitivity analyses to challenge the robustness of the findings. We examined the ATT using inverse probability weighting and nearest neighbor propensity score matching. *P* < 0.05 was considered to be statistically significant.

## Results

### Patients

Data were collected on 29,922 patients with colectomies for colon cancer with or without obstruction (any stage) in the 2013–2015 NSQIP data with ASA class < 5. Of those, 1370 patients were diagnosed with locally advanced colon cancer (T4) non-emergently with ASA class 0-IV, 563 patients underwent LC and 807 patients underwent OC, and these together comprised the study population (Table 1). Patients were predominantly white (71.1%) and non-Hispanic (84.0%). Twenty-nine percent of patients were obese. Most patients had ASA class three (56.0%), and were nonsmokers (82.1%).

**Table 1:** Patient characteristics by laparoscopic versus open surgery, NSQIP 2013–2015.

Patient characteristic	Total (n = 1370)	Laparoscopic surgery (n = 563)	Open surgery (n = 807)
**Sex**			
Male	48	46.7	49
Female	52	53.3	51.1
**Age group (years)**			
< 45	6.5	7.1	6.1
45–54	12.7	13.9	11.9
55–64	21.1	21.5	20.8
65–74	24.4	25	23.9
75 +	35.3	32.5	37.3
**Race**			
White	71.1	70.9	71.3
African American	10.5	9.1	11.5
Other	4.3	1.3	4.3
Unknown	14.1	15.8	12.9
**Hispanic ethnicity**			
No	84	83	84.8
Yes	2.6	3.6	1.9
Unknown	13.4	13.5	13.4
Body mass index*			
≥ 30 kg/m^2^	29	33.8	25.7
< 30 kg/m^2^	71	66.2	74.3
ASA category*			
1	1.8	2.8	1.1
2	33.8	40.7	29
3	56	51.3	59.2
4	8	5	10.2
Unknown	0.4	0.2	0.5
Tumor type*			
T4	14.5	14.4	14.6
T4a	53.3	65.2	45
T4b	32.2	20.4	40.4
**Current smoker**			
Yes	17.9	18.1	17.7
No	82.1	81.9	82.3
Preoperative mechanical bowel preparation*			
Yes	49.9	56.1	45.6
No	37.5	30.9	42
Unknown	12.6	13	12.4
Cancer location*			
Left	36.1	37.8	34.9
Right	43.5	46.2	41.6
Transverse	20.4	16	23.4
AJCC stage*			
II	36	31.6	39
III	63.8	68	60.8
Unknown	0.2	0.4	0.1
Chemotherapy w/i 90 days *			
Yes	5.8	2.3	8.3
No	93.1	96.5	90.7
Unknown	1.1	1.2	1
Diabetes	16.7	16.3	17
Functional health status	4.3	3	5.1
**Chronic kidney disease**			
0	79.2	77.6	16.6
1	17.1	17.8	80.3
2	3.7	4.6	3.1
Anemia*			
Unknown	2.6	3	2.2
No anemia	33.9	41.6	28.5
Mild	47.3	42.8	50.4
Moderate	13	11	14.4
Severe	3.3	1.6	4.5
**Admission year**			
2013	26	23.1	28
2014	30.9	32.2	30
2015	43.1	44.8	42

* p < 0.05; Other race includes American Indian, Alaskan Native, Native Hawaiian, Pacific Islander, or Asian; CI: confidence interval; SD: standard deviation.

### Unmatched outcomes

Of all patients, 45.1% experienced at least one adverse postoperative outcome (Table 2), which was higher in OC (52.2%) versus LC patients (35.0%) (*p* < 0.05). Mortality was 3.1%, which was higher in OC (4.1%) versus LC patients (1.8%) (*p* < 0.05). Ileus was prolonged in OC patients (26.9%) versus LC patients (14.4%) (*p* < 0.05). Blood transfusions were more frequent in OC (22.2%) versus LC patients (11.9%) (*p* < 0.05). Length of stay was an average of 3 days shorter for LC versus OC patients (*p* < 0.05). In LC patients, 31.3% of patients were converted to open surgery. No other differences existed between OC and LC patients (*p* > 0.05).

**Table 2:** Patient outcomes of laparoscopic versus open colectomy, NSQIP 2013–2015.

Patient outcome	Total	Unmatched analysis		Propensity-matched analysis	
	(n = 1370)	Laparoscopic colectomy (n = 563)	Open colectomy (n = 807)	Average treatment effect: LC versus OC (%)	Total number of patients (1:1 match)
**Postoperative**					
Composite outcome (%)**	45.1	35.0*	52.2	− 10.7 (− 16.7; − 4.7)	1106
Mortality (%)	3.1	1.8*	4.1	− 0.8 (− 0.3; 1.1)	1106
Readmission (%)	11.1	9.4	12.3	− 3.3 (− 7.3; 0.5)	1104
Reoperation (%)	5.2	5.3	5.1	0.2 (− 2.6; 3.1)	1106
Sepsis (%)	6.0	5.0	6.7	− 0.2 (− 3.0; 2.6)	1106
Prolonged postoperative ileus (%)	21.8	14.4*	26.9	-11.0 (-15.7; -6.2)	1104
Bleeding requiring blood transfusion (%)	18.0	11.9*	22.2	− 4.3 (− 8.7; 0.0)	1106
Mean length of stay in days mean, SD)	9.4 (8.5)	7.6 (8.1)*	10.7 (8.5)	− 2.1 (− 3.1; − 1.1)	1106
**Peri-operative**					
Mean length of operation in minutes (SD)	180.2 (99.1)	185.2 (90.8)	176.7 (104.5)	14.3 (2.2; 26.5)	1104
Anastomotic leak (%)	3.5	3.2	3.7	0.1 (− 2.1; 2.4)	1102

* p < 0.05 in unadjusted LC (laparoscopic colectomy) versus OC (open colectomy) comparison; **Composite outcome includes readmission, reoperation, wound infection, blood transfusion, prolonged ileus, sepsis, myocardial infarction, deep vein thrombosis, pulmonary embolism, and mortality; SD: standard deviation.

### Propensity-score matched outcomes

The percentage of patients who experienced at least one of the adverse postoperative outcomes was 10.7% (95% CI − 16.7; − 4.7) lower in LC versus OC patients (Table 2). For every nine patients treated with LC, there was one fewer adverse postoperative outcome compared to OC. Prolonged ileus was 11.0% (95% CI − 15.7; − 6.2) lower in LC versus OC patients. The percentage of LC patients requiring blood transfusion was 4.3% (95% CI − 8.7; 0.0) lower in LC versus OC patients. Length of stay was an average of 2 days (95% CI − 3.1; − 1.1) shorter for LC versus OC patients. However, LC patients were in the operating room an average of 14.3 min longer (95% CI 2.2; 26.5). Mortality was similar in both patient groups. No other statistical differences existed between OC and LC patients. For all comparisons, LC and OC patients were well balanced after matching based on the standardized differences as well as the means and variances of the covariates.

### Heterogeneity in LC effectiveness

Postoperative outcomes were similar for both LC and OC among patients with T4a and those with T4b colon cancer (*p* = 0.976) (Table 3). There were no significant differences in length of stay between both types of surgery among T4a and T4b colon cancer patients (*p* = 0.989). Mean length of operation was longer among T4b patients regardless of the type of surgery, and LC resulted in longer average operation time among T4a patients (*p* = 0.005).

**Table 3:** Effect of laparoscopic versus open colectomy by type of T4 tumor, NSQIP 2013–2015.

Patient outcome	T4a patients (n = 675)		T4b patients (n = 264)		p*
	LC	OC	LC	OC	
Composite adverse postoperative outcome (%)	110 (30.6%)	142 (45.1%)	53 (46.9%)	80 (53.0%)	0.976
Mean length of stay in days (SD)	7.1 (8.3)	9.9 (9.2)	9.0 (8.0)	10.5 (7.8)	0.989
Mean length of operation in min (SD)	180.1 (87.8)	144.7 (80.5)	209.6 (97.7)	216.8 (123.8)	**0.005**

* *p* < 0.05 based on propensity score-matched patients comparing T4a versus T4b patients.

LC: laparoscopic colectomy; OC: Open colectomy; SD: standard deviation.

Composite outcome includes readmission, reoperation, wound infection, blood transfusion, prolonged ileus, sepsis, myocardial infarction, deep vein thrombosis, pulmonary embolism, and mortality.

Composite adverse postoperative outcomes between LC and OC were similar regardless of where the tumor was located (ascending, descending, transverse colon) (*p* = 0.397) (Table 4). Mean length of stay was consistently lower for LC versus OC patient, but this was irrespective of where the tumor was located (*p* = 0.930). Mean length of operation was lower among tumors located in the ascending colon versus those located in the descending or transverse colon. Additionally, the difference in operation time between LC and OC differed by location, with LC associated with an increase in operation time for tumors located in the descending colon, a slight reduction in operation time for tumors in the transverse and ascending colon (*p* = 0.001).

**Table 4:** Effect of laparoscopic versus open colectomy by type of tumor location, NSQIP 2013–2015.

Patient outcome	Ascending colon (n = 506)		Descending colon (n = 410)		Transverse colon (n = 188)		p*
	LC	OC	LC	OC	LC	OC	
Composite adverse postoperative outcome (%)	91 (35.8%)	126 (50.0%)	69 (32.7%)	88 (44.2%)	32 (36.8%)	51 (50.5%)	0.397
Mean length of stay in days (SD)	7.4 (7.4)	10.1 (7.6)	7.3 (7.6)	10.2 (8.9)	8.2 (10.6)	10.0 (10.8)	0.930
Mean length of operation in min (SD)	150.5 (66.3)	140.8 (82.4)	234.2 (101.6)	198.2 (109.9)	169.7 (74.2)	182.3 (120.5)	**0.001**

* *p* < 0.05 based on propensity score-matched patients comparing tumor location.

LC: laparoscopic colectomy; OC: Open colectomy; SD: standard deviation.

Composite outcome includes readmission, reoperation, wound infection, blood transfusion, prolonged ileus, sepsis, myocardial infarction, deep vein thrombosis, pulmonary embolism, and mortality.

The composite outcome, length of stay, and length of operation were all similar between OC and LC among obese and nonobese patients (Table 5). While 45.2% of obese patients who underwent OC experienced at least one of the composite outcomes compared to 35.5% of obese patients who underwent LC, this same of pattern of worse composite outcomes when undergoing OC was seen among nonobese patients. Thus, LC was similarly effective compared to OC among obese versus nonobese patients with T4 colon cancer (*p* = 0.295). Mean length of stay was about two days shorter for LC versus OC, but that difference existed for both obese and nonobese patients (*p* = 0.589). The mean length of operation was about 20 min longer in LC versus OC patients, but this difference was similar for both obese and nonobese T4 colon cancer patients (*p* = 0.357).

**Table 5:** Effect of laparoscopic versus open colectomy by obesity, NSQIP 2013–2015.*

Patient outcome	Obese patients (n = 352)		Nonobese patients (n = 752)		p**
	LC	OC	LC	OC	
Composite adverse postoperative outcome (%)	66 (35.5%)	79 (47.6)	126 (34.4%)	186 (48.2)	0.295
Mean length of stay in days (SD)	7.4 (7.7)	10.8 (10.6)	7.5 (8.3)	9.8 (7.8)	0.589
Mean length of operation in min (SD)	200.8 (95.3)	184.9 (115.3)	177.7 (88.2)	162.2 (97.8)	0.357

*Obese: ≥ 30 kg/m^2^; ** *p* < 0.05 based on propensity score-matched patients comparing obese versus nonobese patients. LC: laparoscopic colectomy; OC: Open colectomy; SD: standard deviation.

Composite outcome includes readmission, reoperation, wound infection, blood transfusion, prolonged ileus, sepsis, myocardial infarction, deep vein thrombosis, pulmonary embolism, and mortality.

Our findings were generally robust with respect to different methods of propensity score matching and inclusion of hand-assisted approach in LC (online Tables 1–4).

## Discussion

This is the first and largest study to examine differences in effectiveness of LC in various types of locally advanced (T4) colon cancer patients. In matched analysis, LC patients had better overall outcomes and shorter lengths of stay compared with OC patients. Studies to date comparing LC and OC in T4 colon cancer patients consist predominantly of single-institution studies and one meta-analysis of five small studies, resulting in unstable estimates of many patient outcomes^13–17^.

T4b tumors are technically more demanding than T4a colon tumors. Our results show that LC is less likely to be used in T4b than T4a cancers, due to possible involvement of other organs requiring multi-visceral resection. We also observed no statistical difference in outcomes using data of over 900 T4a and T4b colon cancer patients, suggesting that LC can be used safely in T4b patients. Only two studies^15,26^ were identified as part of a recent meta-analysis focused on patient outcomes using LC in T4b colon cancers^18^. Neither study observed differences in overall survival between the LC and OC groups, but the power to detect clinically important differences was low because there were fewer than 100 T4b patients in both studies combined.

LC of transverse colon cancers is more challenging than cancers at other locations in the colon because of anatomical constraints^24^. Small studies that excluded locally advanced colon cancers showed few differences between LC and OC in transverse colon cancers^22–24^. Our results extend these findings to suggest that LC can be used safely and effectively in locally advanced cancer, even those located in the transverse colon.

Older studies have argued that obesity could be a contra-indication to LC because of technically difficulties^20,21^. However, our results show that obese patients were more likely to receive LC than nonobese patients during 2013–2015. While LC appears to result in similar patient outcomes in obese and nonobese colon cancer patients^33^, T4 colon cancer patients were not included. Small studies with fewer than 50 patients showed similar outcomes of LC in obese and nonobese patients who receive colon resection for various reasons^34^. Our finding based on 1370 patients now confirm these results to show similar outcomes of LC in obese and nonobese T4 patients, suggesting that there is no reason for obese T4 colon cancer patients not to undergo LC.

A strength of our study is its inclusion of patient outcomes from multiple hospitals reporting on many different types of outcomes. Our matched propensity-score analysis maximized causal inferences^35^. High-quality observational studies can provide information on treatment effectiveness in subpopulations^36^, because it is unlikely that a randomized trial will be conducted to determine which patients benefit from LC.

We also recognize some limitations of our findings. Some patient outcomes were relatively infrequent. Therefore, we constructed a composite outcome, which is less likely to reflect this limitation. In addition, we were unable to control for characteristics of hospitals (e.g., enhanced recovery after surgery), surgeons (e.g., volume), or tumors (e.g., resection margins, lymph nodes reported) in the propensity score because of confidentiality concerns with releasing this data. In addition, the similarity in patient outcomes between LC and OC may be due to more skilled laparoscopists at facilities with higher volume. Thus, the potential for selection bias remains present. Furthermore, we recognize that the NSQIP typically includes data from larger hospitals and is not a nationally representative sample. Finally, we recognize that the effectiveness of LC may vary within the large range of BMIs that is included in our definition of obesity (BMI ≥ 30 kg/m^2^)^37^, but the sample size was insufficient to examine LC effectiveness in patients with BMI ≥ 40 kg/m^2^.

Thus, T4 colon cancer patients, including obese patients, those with T4b colon cancer, and those with cancer located in the transverse colon, can safely receive LC based on their lower rates of adverse outcomes.

## Supplementary Information

Supplementary Information.
