# Supplementary material for: Differences in effectiveness and use of laparoscopic surgery in locally advanced colon cancer patients
Source: Sci Rep. 2021 May 11;11:10022. doi: 10.1038/s41598-021-89554-0 (PMC8113575; doi:10.1038/s41598-021-89554-0)
Supplement: Supplementary file 1 — Supplementary Information. [file 41598_2021_89554_MOESM1_ESM.docx]

**SENSITIVITY ANALYSES – online tables**

eTable 1. Patient outcomes of laparoscopic colectomy INCLUDING HAND ASSISTANCE versus open colectomy, NSQIP 2013-2015.

|  | Propensity-matched analysis | |
| --- | --- | --- |
| Patient outcome | Average treatment effect: LC versus OC (%) | Total number of patients (1:1 match) |
| Postoperative |  |  |
| Composite outcome (%)* | -13.7 (-18.9; -8.5) | 1,106 |
| Mortality (%) | -0.8 (-2.6; 1.1) | 1,106 |
| Readmission (%) | -2.1 (-5.5; 1.3) | 1,104 |
| Reoperation (%) | 1.1 (-1.6; 3.8) | 1,106 |
| Sepsis (%) | 0.6 (-1.7; 3.0) | 1,106 |
| Prolonged postoperative ileus (%) | -11.6 (-15.8; -4.4) | 1,104 |
| Bleeding requiring blood transfusion (%) | -3.8 (-7.4; -0.2) | 1,106 |
| Mean length of stay in days (mean, SD) | -2.2 (-3.1; -1.4) | 1,106 |
| Peri-operative |  |  |
| Mean length of operation in minutes (SD) | 11.8 (1.3; 22.3) | 1,104 |
| Anastomotic leak (%) | -0.2 (-1.9; 1.6) | 1,102 |

* Composite outcome includes readmission, reoperation, wound infection, blood transfusion, prolonged ileus, sepsis, myocardial infarction, deep vein thrombosis, pulmonary embolism, and mortality; SD: standard deviation

eTable 2. Effect of laparoscopic versus open colectomy by type of T4 tumor, NSQIP 2013-2015.

|  | T4a patients (n=828) | |  | T4b patients (n=507) | |  | p* |  |
| --- | --- | --- | --- | --- | --- | --- | --- | --- |
| Patient outcome | LC | OC |  | LC | OC |  |  |  |
| Composite adverse postoperative outcome (%) | 148 (31.0%) | 163 (46.6%) |  | 81 (43.8%) | 185 (57.4%) |  | 0.769 |  |
| Mean length of stay in days (SD) | 7.2 (8.0) | 10.2 (9.1) |  | 8.7 (7.4) | 10.9 (7.3) |  | 0.363 |  |
| Mean length of operation in min (SD) | 1.70.5 (81.1) | 143.4 (79.1) |  | 212.8 (109.8) | 212.7 (116.1) |  | 0.052 |  |

* p value based on propensity score-matched patients comparing T4a versus T4b patients.

LC: laparoscopic colectomy; OC: Open colectomy; SD: standard deviation

Composite outcome includes readmission, reoperation, wound infection, blood transfusion, prolonged ileus, sepsis, myocardial infarction, deep vein thrombosis, pulmonary embolism, and mortality

eTable 3. Effect of laparoscopic versus open colectomy by type of tumor location, NSQIP 2013-2015.

|  | Ascending colon (n=686) | |  | Descending colon (n=551) | |  | Transverse colon (n=333) | | p* |
| --- | --- | --- | --- | --- | --- | --- | --- | --- | --- |
| Patient outcome | LC | OC |  | LC | OC |  | LC | OC |  |
| Composite adverse postoperative outcome (%) | 126 (34.8) | 170 (52.5%) |  | 98 (36.2) | 141 (50.4%) |  | 50 (32.9) | 100 (55.2%) | 0.1957 |
| Mean length of stay in days (SD) | 7.0 (6.6) | 10.4 (7.7) |  | 8.1 (8.2) | 10.7 (8.5) |  | 7.8 (8.8) | 11.0 (9.8) | 0.516 |
| Mean length of operation in min (SD) | 148.3 (61.7) | 142.8 (81.5) |  | 230.9 (110.5) | 212.3 (112.7) |  | 166.2 (70.7) | 183.7 (110.4) | **0.014** |

* p value based on propensity score-matched patients comparing tumor location.

LC: laparoscopic colectomy; OC: Open colectomy; SD: standard deviation

Composite outcome includes readmission, reoperation, wound infection, blood transfusion, prolonged ileus, sepsis, myocardial infarction, deep vein thrombosis, pulmonary embolism, and mortality

eTable 4. Effect of laparoscopic versus open colectomy by obesity, NSQIP 2013-2015.*

|  | Obese patients (n=425) | |  | Nonobese patients (n=1145) | |  | p** |
| --- | --- | --- | --- | --- | --- | --- | --- |
| Patient outcome | LC | OC |  | LC | OC |  |  |
| Composite adverse postoperative outcome (%) | 79 (35.1%) | 98 (49.0%) |  | 195 (34.8%) | 313 (53.5%) |  | 0.425 |
| Mean length of stay in days (SD) | 7.2 (6.9) | 10.8 (10.4) |  | 7.7 (7.9) | 10.6 (7.8) |  | 0.372 |
| Mean length of operation in min (SD) | 204.0 (105.2) | 188.5 (115.6) |  | 170.7 (83.2) | 173.1 (100.7) |  | **0.013** |

* Obese: ≥30 kg/m^2^; ** p value based on propensity score-matched patients comparing obese versus nonobese patients. LC: laparoscopic colectomy; OC: Open colectomy; SD: standard deviation

Composite outcome includes readmission, reoperation, wound infection, blood transfusion, prolonged ileus, sepsis, myocardial infarction, deep vein thrombosis, pulmonary embolism, and mortality
